# A review of optical coherence tomography angiography (OCTA)

**DOI:** 10.1186/s40942-015-0005-8

**Published:** 2015-04-15

**Authors:** Talisa E de Carlo, Andre Romano, Nadia K Waheed, Jay S Duker

**Affiliations:** 1 New England Eye Center and Tufts Medical Center, Tufts University, 260 Tremont Street, Biewend Building, 9 - 11th Floor, Boston, MA 02116 USA; 2 Department of Electrical Engineering and Computer Science, and Research Laboratory of Electronics, Massachusetts Institute of Technology, Cambridge, MA 02139 USA; 3 Department of Ophthalmology, Federal University of São Paulo, Escola Paulista de Medicina, São Paulo, Brazil; 4 Retina Service, Neovista Eye Center, Americana, Brazil

**Keywords:** Age-related macular degeneration, Diabetic retinopathy, Fluorescein angiography, Glaucoma, Indocyanine angiography, Optical coherence tomography angiography, Optic disc, Retina, Retinal vessel occlusion

## Abstract

Optical coherence tomography angiography (OCTA) is a new, non-invasive imaging technique that generates volumetric angiography images in a matter of seconds. This is a nascent technology with a potential wide applicability for retinal vascular disease. At present, level 1 evidence of the technology’s clinical applications doesn’t exist. In this paper, we introduce the technology, review the available English language publications regarding OCTA, and compare it with the current angiographic gold standards, fluorescein angiography (FA) and indocyanine green angiography (ICGA). Finally we summarize its potential application to retinal vascular diseases. OCTA is quick and non-invasive, and provides volumetric data with the clinical capability of specifically localizing and delineating pathology along with the ability to show both structural and blood flow information in tandem. Its current limitations include a relatively small field of view, inability to show leakage, and proclivity for image artifact due to patient movement/blinking. Published studies hint at OCTA’s potential efficacy in the evaluation of common ophthalmologic diseases such age related macular degeneration (AMD), diabetic retinopathy, artery and vein occlusions, and glaucoma. OCTA can detect changes in choroidal blood vessel flow and can elucidate the presence of choroidal neovascularization (CNV) in a variety of conditions but especially in AMD. It provides a highly detailed view of the retinal vasculature, which allows for accurate delineation of the foveal avascular zone (FAZ) in diabetic eyes and detection of subtle microvascular abnormalities in diabetic and vascular occlusive eyes. Optic disc perfusion in glaucomatous eyes is notable as well on OCTA. Further studies are needed to more definitively determine OCTA’s utility in the clinical setting and to establish if this technology may offer a non-invasive option of visualizing the retinal vasculature in detail.

## Introduction

Optical coherence tomography angiography (OCTA) is a new non-invasive imaging technique that employs motion contrast imaging to high-resolution volumetric blood flow information generating angiographic images in a matter of seconds. OCTA compares the decorrelation signal (differences in the backscattered OCT signal intensity or amplitude) between sequential OCT b-scans taken at precisely the same cross-section in order to construct a map of blood flow. Axial bulk motion from patient movement is eliminated so sites of motion between repeated OCT b-scans represent strictly erythrocyte movement in retinal blood vessels [1-4].

OCTA requires higher imaging speeds than most currently available OCT systems can provide in order to obtain a densely sampled volume. Conventional OCT device scanning speeds would result in too much trade-off between decreased field of view, lower image quality, and greatly increased scanning time.

### Comparing OCTA with FA and ICGA

Fluorescein angiography (FA) and indocyanine green angiography (ICGA) are both invasive test that require intravenous administration of dye and imaging up to 10–30 minutes [5-9]. They provide two-dimensional image sets that allow for dynamic visualization of blood flow with a wide field of view. Therefore, patterns of dye leakage, pooling, and staining can be appreciated and are well-documented in the literature [10]. FA remains the gold standard for the detection of choroidal neovascularization (CNV), as well as retinal neovascularization such as neovascularization of the disc (NVD) and neovascularization elsewhere (NVE) [11-13]. However, retinal pathology can be obscured by this leakage as well as hemorrhage or media opacities, and localization of the depth of the lesion and size delineation of neovascularization can be difficult due to dye leakage and poor stereopsis, and because the imaging modalities are not depth resolved. As a result, segmentation of different layers is not routinely possible with FA or ICGA. Therefore, identification of the axial location of pathology requires an understanding of patterns of blockage and leakage [10]. For example, differentiation between type 1 CNV, which is found between the retinal pigment epithelium (RPE) and Bruch’s membrane, and type 2 CNV, which is found in the subretinal space above the RPE, requires understanding that the RPE blocks underlying fluorescence so type 1 CNV requires a larger amount of dye to accumulate before hyperfluorescence is apparent [14].

FA and ICGA have other drawbacks that can limit their widespread use. Since they are invasive, relatively expensive, and time-consuming, they are not ideal techniques to use on a regular basis in a busy clinical setting. Although considered safe, the dyes pose risks ranging from nausea to allergic reactions, including anaphylaxis in rare instances. Aside from allergic reactions of which the likelihood increases with frequency of use, indocyanine green dye is contraindicated in pregnancy and kidney disease [15-17]. For the evaluation of patients requiring frequent follow-up exams or of those that may not tolerate injection of intravenous dye, a rapid non-invasive technique to visualize retinal and choroidal vessels would be beneficial.

**Figure 1:**
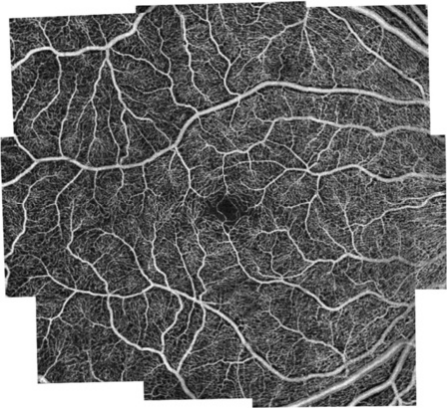
**OCTA Wide-Field Montage of a Normal Eye.** Optical coherence tomography angiography (OCTA) wide-field montage of the normal right eye of a 56 year old Caucasian man. Images were acquired using the Angiovue software of the RTVue XR Avanti (Optovue, Inc., Fremont, CA) and montaged using Adobe Photoshop (San Jose, CA). This allows for a larger field of view much like fluorescein and indocyanine green angiography while maintaining improved resolution (de Carlo TE *et al*., unpublished data in review).

OCTA in comparison is a non-invasive technique that acquires volumetric angiographic information without the use of dye. Each three-dimensional scan set takes approximately six seconds to obtain. The en-face images (OCT angiograms) can then be scrolled outward from the internal limiting membrane (ILM) to the choroid to visualize the individual vascular plexus and segment the inner retina, outer retina, choriocapillaris, or other area of interest. The en-face acquisition areas currently range from 2 × 2 mm to 12 × 12 mm with the scan quality greatly decreased with a widened field of view since the same number of OCT b-scans is used for all scanning areas. The 12 x 12 mm scan is only available on research prototypes. The 3 × 3 mm OCT angiograms appear to be higher resolution than the currently available FA/ICGA images, and a study by Matsunaga *et al.* deduced that they were at least equivalent in showing important vascular detail [18]. Use of the montage technique allows for a larger field of view much like FA/ICGA while maintaining this improved resolution (Figure 1; de Carlo TE *et al*., unpublished data in review). Carl Zeiss, Inc (Carl Zeiss Meditec, Dublin, CA) is developing an automatic wide-field montage software, which employs motion tracking to track the eyes and stitch images together.

OCTA provides flow information at a fixed point in time. Although leakage is not appreciable, exact delineation and size measurements can be performed for pathology such as CNV (de Carlo TE *et al.,* unpublished data in review) [19]. This is especially useful for identification of type 1 CNV where localization is inferential and therefore may be inaccurate with FA/ICGA. Retinal blood flow on OCTA can be obscured by hemorrhage as this decreases the ability of light to penetrate into the deeper layers of the eye.

OCTA provides both structural and functional (i.e. blood flow) information in tandem. The “corresponding” OCT b-scans can be co-registered with the simultaneous OCT angiograms so the operator is able to scroll through the OCT angiogram like a cube scan. As a result, the precise location of pathology can be viewed on the corresponding OCT b-scans. The axial resolution of the corresponding OCT b-scans are lower quality than the typical highly-sampled line scans and are similar to the resolution of individual OCT b-scans within a volumetric cube scan. Both the retinal and the choroidal microvasculature can be visualized using OCTA while FA is used for seeing the retinal vessels and ICGA is more ideal for imaging the choroid.

Using the present technology, OCTA is more prone to artifact than FA or ICGA. The larger retinal vessels cause a “ghost image” referred to as a shadow artifact, when segmenting deeper layers, especially the outer retina. This can make it more difficult to appreciate the presence of abnormal vasculature in the deeper layers. Because OCTA uses the principle that movement in the back of the eye represents blood flow, it is prone to motion artifact. White lines (representing decorrelation signal over the entire b-scan) appear in areas of bulk patient movement such as when the patient loses fixation or moves. Conversely, blinks appear as a black line across the OCT angiogram because the OCT signal is blocked from reaching the retina and the software, therefore, detects no movement. Although erythrocytes should be the only moving object in the retina, some non-vascular structures such as fine tissue may also cause a decorrelation signal, especially if the patient is moving. For example, the edges of a retinal pigment epithelial detachment (RPED) often show up on OCTA as white noise artifact in cases of increased patient movement. It is postulated that because the RPE is a fine structure, in areas of disruption such as a RPED, it can presumably move and therefore be detected on the OCT angiogram.

On the other hand, OCTA can also miss areas of slow blood flow such as in microaneurysms or fibrotic CNV. Since OCTA relies on change between consecutive b-scans, it will detect flow only above a minimum threshold, the slowest detectable flow, which is determined by the time between the two sequential OCT b-scans. Lesions that have flow below the slowest detectable flow would therefore not be visualized using this imaging technique. Increasing the time between consecutive OCT b-scans could allow for increased flow detection but would offer a trade-off due to increased movement artifact. One of the advantages of a higher speed system is that multiple volumetric sets can be obtained at each cross-section so the threshold can be altered later by selecting different time frames between the OCT b-scans to determine the optimal image quality. Therefore if a low-flow vessel is undetectable by using the first and second OCT b-scans at a given cross section, the image may be processed using the first and third OCT b-scans to increase the time between the OCT b-scans thereby decreasing the minimum threshold.

A couple of publications have qualitatively compared OCTA with FA. Spaide *et al.* described the peripapillary retinal vascular layers in 12 normal eyes, finding that OCTA provided improved visualization of all the vascular layers including the radial peripapillary and deep capillary networks that were not well-distinguished on FA [3]. OCTA imaging of the perifoveal region was reported by Matsunaga *et al.*, demonstrating that the ability to see the normal retinal vasculature was equivalent to that of FA [18].

## Review

### OCTA of normal eyes

**Figure 2:**
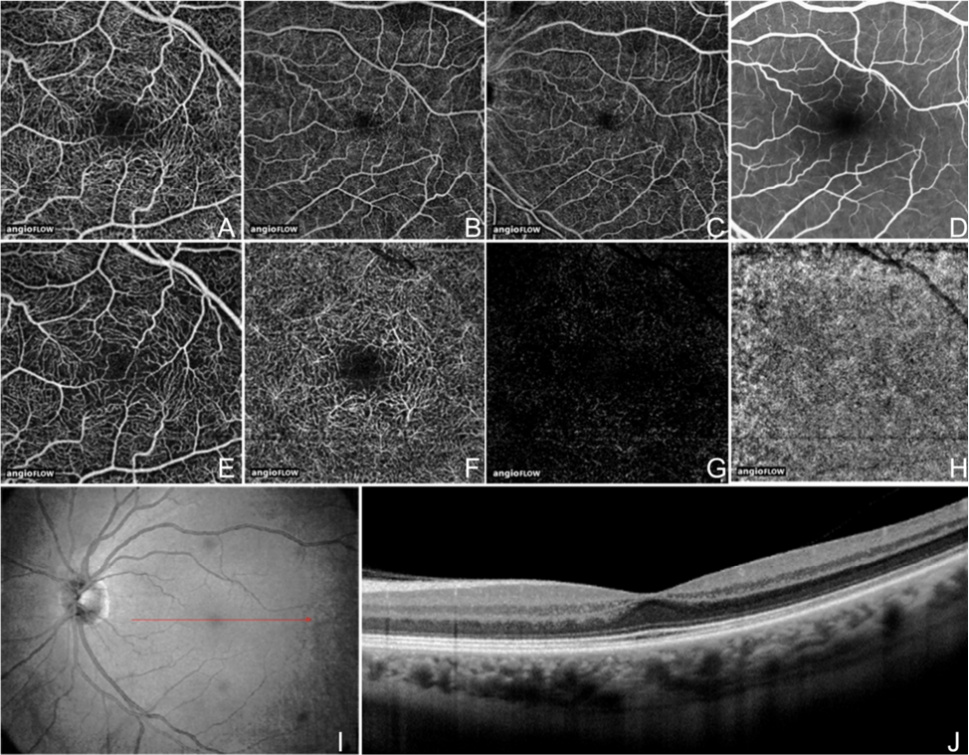
**OCT Angiogram Fields of View and Segmentation Layers on Angiovue.** The normal left eye of a 56 year old Caucasian man using the Angiovue optical coherence tomography angiography (OCTA) software of the RTVue XR Avanti (Optovue, Inc., Fremont, CA). **(A)** Full-thickness (internal limiting membrane to Bruch’s membrane) 3 x 3 mm OCT angiogram. **(B)** Full-thickness 6 x 6 mm OCT Angiogram. **(C)** Full-thickness 8 x 8 mm OCT Angiogram. **(D)** Fluorescein angiography cropped to approximately 8 x 8 mm or 30 degrees demonstrates less capillary detail than A-C. **(E)** 3 x 3 mm OCT angiogram of the “Superficial” inner retina. **(F)** 3 x 3 mm OCT angiogram of the “Deep” inner retina. **(G)** 3 x 3 mm OCT angiogram of the outer retina shows absence of vasculature. The white represents noise. **(H)** 3 x 3 mm OCT angiogram of the choriocapillaris is generally homogenous. There is black shadowing from retinal vessels. **(I)** En-face intensity OCT image. **(J)** Highly-sampled OCT b-scan image.

The most widely available prototype OCTA system is the AngioVue software of the RTVue XR Avanti spectral-domain OCT (SD-OCT) (Optovue, Inc, Fremont, CA), which uses a split-spectrum amplitude decorrelation angiography (SSADA) algorithm. The device obtains volumetric scans of 304 × 304 A-scans at 70,000 A-scans per second in approximately 3.0 seconds. The software offers the option of 2 × 2 mm, 3 × 3 mm, 6 × 6 mm, and 8 × 8 mm OCT angiograms (Figure 2A-C) and automated segmentation of these full-thickness retinal scans into the “superficial” and “deep” inner retinal vascular plexuses, outer retina, and choriocapillaris (Figure 2E-H). The OCT angiogram segmentation of the superficial inner retina contains a projection of the vasculature in the retinal nerve fiber layer (RNFL) and ganglion cell layer (GCL) (Figure 2E). The deep inner retina OCT angiogram segmentation shows a composite of the vascular plexuses at the border of the inner plexiform layer (IPL) and inner nuclear layer (INL) and the border of the INL and outer plexiform layer (OPL) (Figure 2F).

**Figure 3:**
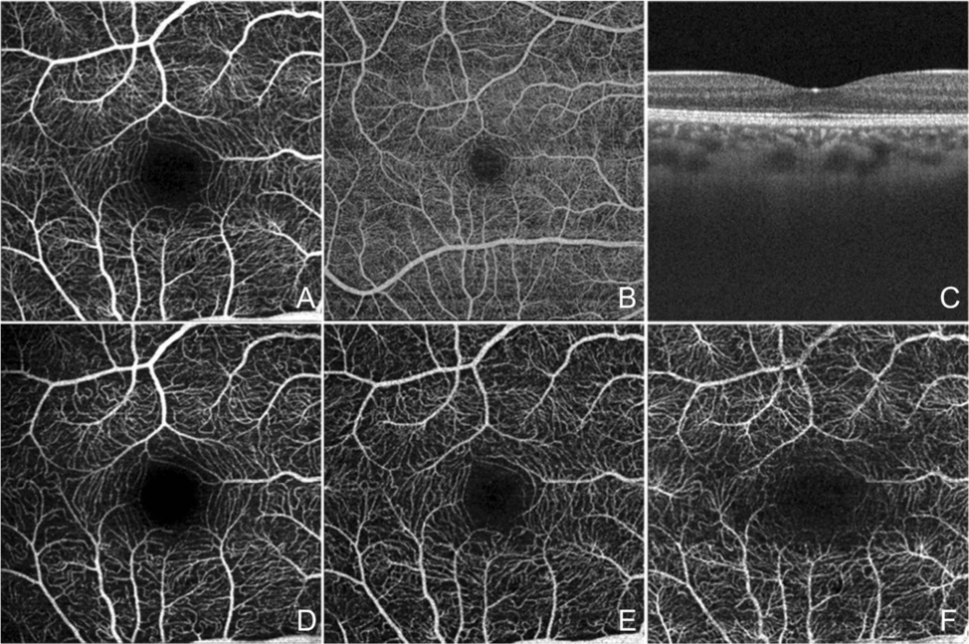
**OCT Angiogram Fields of View and Segmentation Layers on the SS-OCT Protype.** The normal right eye of a 26 year old Caucasian woman using a prototype swept source optical coherence tomography angiography (OCTA) system (Department of Electrical Engineering and Computer Science and Research Laboratory of Electronics, Massachussetts Insitute of Technology, Cambridge, MA). **(A)** Full-thickness (internal limiting membrane to Bruch’s membrane) 3 x 3 mm OCT angiogram. **(B)** Full-thickness 6 x 6 mm OCT angiogram. **(C)** Corresponding OCT b-scan. **(D)** 3 x 3 mm OCT angiogram of the retinal nerve fiber layer plexus of the inner retina. **(E)** 3 x 3 mm OCT angiogram of the ganglion cell layer plexus of the inner retina. **(F)** 3 x 3 mm OCT angiogram of the “deep” inner retina.

The OCTA prototype with the fastest acquisition rate was developed by the Massachusetts Institute of Technology using a swept-source OCT (SS-OCT) device (Department of Electrical Engineering and Computer Science and Research Laboratory of Electronics, Massachussetts Institute of Technology, Cambridge, MA). This ultra-high speed prototype employs a vertical cavity surface emitting laser (VCSEL) operating at 1060 nm wavelength which allows increased light penetration into pigmented tissues and improved choroidal blood flow visualization compared to the light source used in SD-OCT. The SS-OCTA system obtains scans of 500 × 500 A-scans at 400,000 A-scans per second in approximately 3.8 seconds. This ultra-high speed allows for imaging of wider fields of view. The prototype can be manipulated to obtain OCT angiograms up to 12 × 12 mm, however, it is most commonly used to create 3 × 3 mm and 6 × 6 mm OCT angiograms of great detail (Figure 3A-B). Full-thickness scans are manually segmented into the superficial (plexus at the RNFL), intermediate (plexus at the GCL), and deep (plexuses at IPL/INL and INL/OPL borders) inner retinal vascular plexuses, outer retina, choriocapillaris, and choroidal layers (Figure 3D-F). Using this OCTA system, the choriocapillaris and choroidal vessels were described in normal eyes by Choi *et al* [2].

### OCTA of dry (Non-Neovascular) AMD

**Figure 4:**
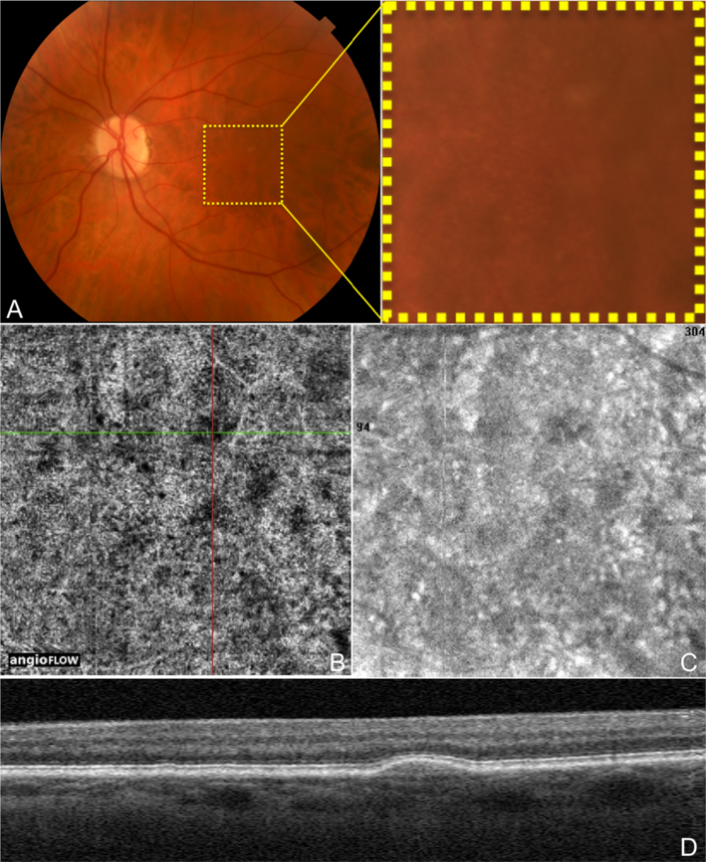
**OCTA and Color Fundus Photo of Drusen in Non-Neovascular AMD.** The left eye of a 72 year old Caucasian man with non-neovascular age-related macular degeneration using the Angiovue optical coherence tomography angiography (OCTA) software of the RTVue XR Avanti (Optovue, Inc., Fremont, CA). **(A)** Color fundus photo zoomed in to an approximately 3 x 3 mm area centered at the macula showing hard and soft drusen. **(B)** 3 x 3 mm OCT angiogram of the choriocapillaris centered at the macula as in A. The green and red lines represent the x and y axis OCT b-scans respectively which cross at a soft druse demonstrating an area of decreased signal in the choriocapillaris underlying the druse. **(C)** 3 x 3 mm en-face structural OCT of the choriocapillaris centered at the macula as in A-B. This image was simultaneously obtained during the same scan as the OCT angiogram in B. This structural OCT is still able to show the choriocapillaris changes at the location of the soft drusen in B, but detail is overall limited. **(D)** Corresponding x axis OCT b-scan at the cross-section demonstrated by the green line in B showing the soft druse. The corresponding OCT b-scans were simultaneously obtained during the same scan as the OCT angiogram in B.

**Figure 5:**
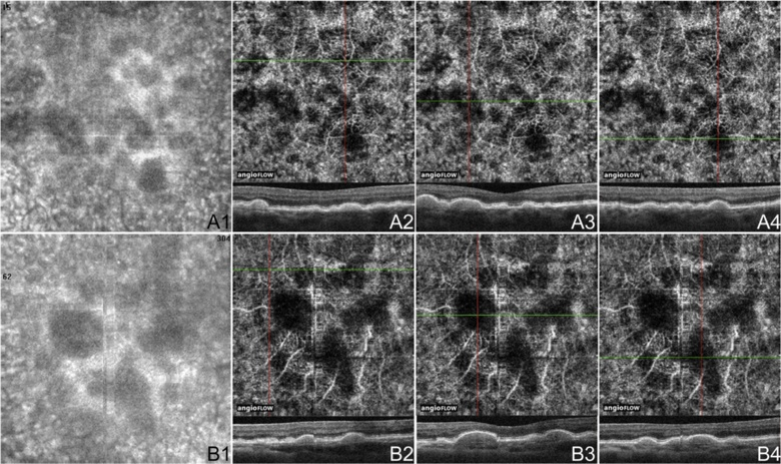
**OCTA of Drusen in Non-Neovascular AMD Cases. (A)** 3 x 3 mm en-face images of the right eye of a 74 year old Caucasian man with non-neovascular age-related macular degeneration (AMD) using the Angiovue optical coherence tomography angiography (OCTA) software of the RTVue XR Avanti (Optovue, Inc., Fremont, CA). **(A1)** En-face structural OCT demonstrating areas of choriocapillaris alteration. **(A2-4)** OCT angiograms of the choriocapillaris and corresponding x-axis OCT b-scans at the cross-sections shown by the green line of the OCT angiograms. The three soft drusen shown are associated with areas of decreased signal in the choriocapillaris, which could indicate flow impairment. **(B)** 3 x 3 mm en-face images of the left eye of an 80 year old Asian woman with non-neovascular AMD using the Angiovue OCTA software of the RTVue XR Avanti (Optovue, Inc., Fremont, CA). **(B1)** En-face structural OCT demonstrating areas of choriocapillaris changes. **(B2-4)** OCT angiograms of the choriocapillaris and corresponding x-axis OCT b-scans at the cross-sections shown by the green line of the OCT angiograms. The druse in B2 is not associated with choriocapillaris loss. The other two soft drusen shown correspond to areas of decreased signal in the choriocapillaris, which could indicate flow impairment.

**Figure 6:**
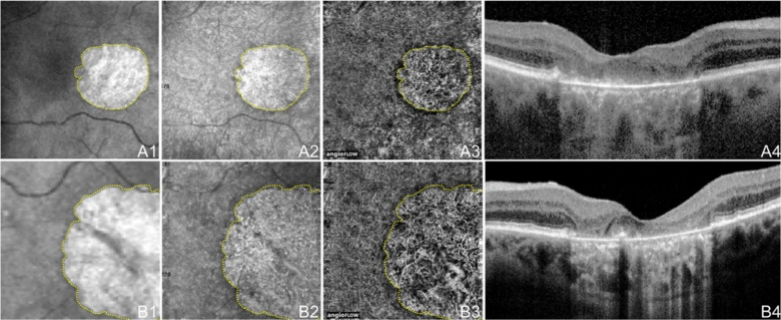
**OCTA of GA in Non-Neovascular AMD.** 71 year old Caucasian man with geographic atrophy (GA) due to non-neovascular age-related macular degeneration using the Angiovue optical coherence tomography angiography (OCTA) software of the RTVue XR Avanti (Optovue, Inc., Fremont, CA). **(A)** 6 x 6 mm en-face images of the right eye. **(A1)** En-face structural OCT at the level of the RPE demonstrating GA. The area of GA is circumscribed in yellow, which is overlaid over the images in A2 and A3. **(A2)** En-face structural OCT at the level of the choriocapillaris demonstrating alteration in a similar area as the GA. **(A3)** OCT angiogram at the level of the choriocapillaris demonstrating flow impairment in a similar area as the GA. Larger choroidal vessels have been push inward into the area of choriocapillaris alteration so are seen in this 10micrometer slice. Detail is greatly improved over that of the en-face structural OCT. **(A4)** Corresponding OCT b-scan shows the loss of RPE causing increased intensity below Bruch’s membrane which is characteristic of GA. **(B)** 3 x 3 mm en-face images of the left eye. **(B1)** En-face structural OCT at the level of the RPE demonstrating GA. The area of GA is circumscribed in yellow, which is overlaid over the images in B2 and B3. **(B2)** En-face structural OCT at the level of the choriocapillaris demonstrating alteration in a similar area as the GA. **(B3)** OCT angiogram at the level of the choriocapillaris demonstrating flow impairment in a similar area as the GA. Larger choroidal vessels have been push inward into the area of choriocapillaris alteration so are seen in this 10micrometer slice. Detail is greatly improved over that of the en-face structural OCT. **(B4)** Corresponding OCT b-scan shows the loss of RPE causing increased intensity below Bruch’s membrane which is characteristic of GA.

Dry age-related macular degeneration (AMD) is characterized by drusen, pigmentary changes, and photoreceptor and RPE loss, called geographic atrophy (GA). Decreased foveolar choroidal blood flow is associated with AMD and increased drusen extent, and it has been hypothesized that the choroidal blood flow may predict disease progression [14]. Choi *et al.* (unpublished data, presented in part at the Association for Research in Vision and Ophthalmology annual meeting, May 2014, Orlando, Florida) demonstrated OCTA findings in dry AMD. Areas of impaired choriocapillaris flow typically extended beyond the borders of the GA. Eyes with dry AMD were shown to have a generalized decrease in choriocapillaris density, which was sometimes associated with drusen. Figures 4 and 5 demonstrate discrete areas of decreased signal at the choriocapillaris level below many but not all drusen in three eyes. These areas of alteration did not appear to be due to shadowing (from material in the drusen), and some choroidal vessels were appreciated below these areas. However, further studies would be necessary to determine if the choriocapillaris changes associated with the drusen are true areas of flow impairment. Choriocapillaris flow alterations are also shown in two eyes along the border of GA in Figure 6.

### OCTA of wet (Neovascular) AMD

**Figure 7:**
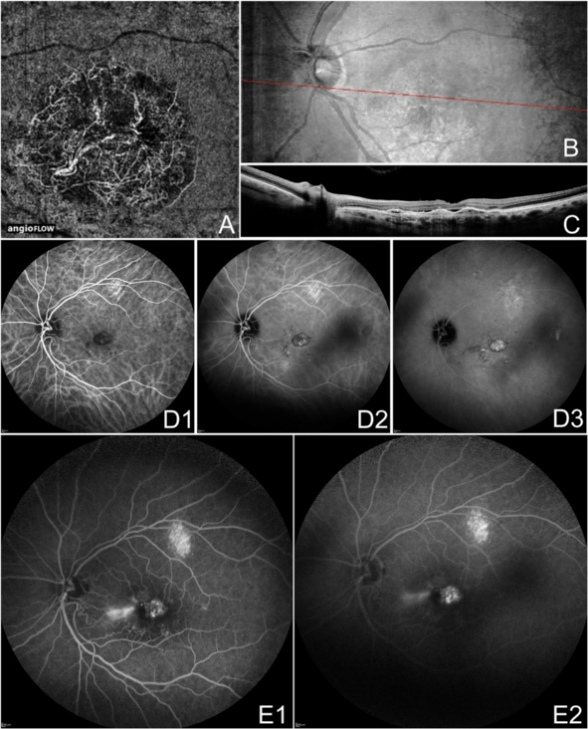
**OCTA and FA/ICGA of CNV in Neovascular AMD.** The left eye of a 67 year old Caucasian man with choroidal neovascularization (CNV) due to neovascular age-related macular degeneration using the Angiovue optical coherence tomography angiography (OCTA) software of the RTVue XR Avanti (Optovue, Inc., Fremont, CA). **(A)** 6 x 6 mm OCT angiogram segmented so both the choriocapillaris and the outer retina are shown. A circular net of abnormal vessels are shown surrounded by relatively homogenous choriocapillaris. The abnormal vessels exist both below and above Bruch’s membrane (in the outer retina). **(B)** En-face structural OCT with a red line corresponding to the highly-sampled OCT b-scan in C. **(C)** 12 mm highly sampled OCT b-scan through the fovea demonstrates a large retinal pigment epithelial detachment, subretinal fluid, disruption of Bruch’s membrane, and hyper-reflective material characteristic of CNV. **(D)** Indocyanine green angiography early, intermediate, and late frames show increasing hyper-fluorescence and pooling of dye in the CNV. **(E)** Fluorescein angiography intermediate and late frames show increasing hyper-fluorescence and pooling of the CNV.

**Figure 8:**
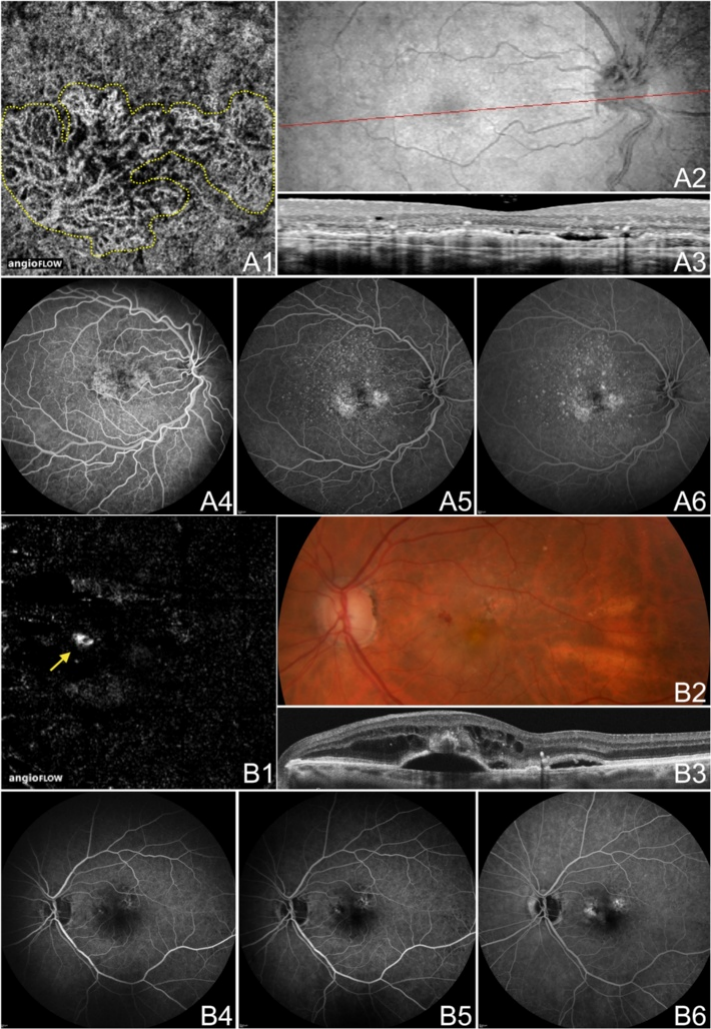
**OCTA and FA of CNV in Neovascular AMD. (A)** The right eye of a 63 year old Caucasian man with choroidal neovascularization (CNV) due to neovascular age-related macular degeneration (AMD) using the Angiovue optical coherence tomography angiography (OCTA) software of the RTVue XR Avanti (Optovue, Inc., Fremont, CA). **(A1)** 3 x 3 mm OCT angiogram segmented so both the choriocapillaris and the outer retina are shown. Two nets of abnormal vessels are shown surrounded by relatively homogenous choriocapillaris. The abnormal vessels exist both below and above Bruch’s membrane (in the outer retina). **(A2-3)** En-face structural OCT with a red line corresponding to a 12 mm highly sampled OCT b-scan (cropped to 3 mm) through the macula. The OCT b-scan demonstrates a retinal pigment epithelial detachment (RPED), subretinal fluid, an intraretinal cyst, and hyper-reflective material characteristic of CNV. **(A4-6)** Fluorescein angiography (FA) early, intermediate, and late frames showing increasing hyper-fluorescence and staining of the CNV. **(B)** The left eye of an 89 year old Caucasian woman with CNV type three (retinal angiomatous proliferation, RAP) due to neovascular AMD using the Angiovue OCTA software of the RTVue XR Avanti (Optovue, Inc., Fremont, CA). **(B1)** 6 x 6 mm OCT angiogram segmented at the outer retina showing a round RAP lesion (yellow arrow). A feeder vessel from a retinal vessel was noted (not shown). **(B2)** Color fundus photo demonstrating hemorrhage in the region of the RAP lesion. **(B3)** 6 mm highly sampled OCT b-scan through the macula shows subretinal and intraretinal fluid and a round ball of hyper-reflective tissue above a serous RPED. **(B4-6)** FA early, intermediate, and late frames showing increasing hyper-fluorescence and pooling in the CNV.

**Figure 9:**
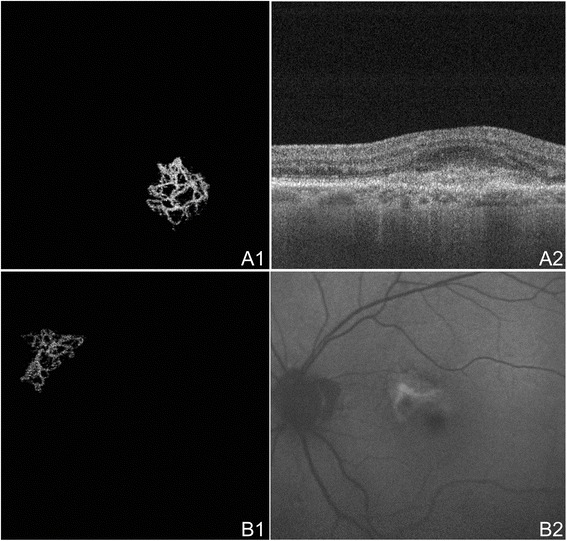
**OCTA of CNV in Neovascular AMD. (A)** The left eye of an 89 year old Caucasian man with choroidal neovascularization (CNV) due to neovascular age-related macular degeneration (AMD) using the swept source optical coherence tomography angiography (OCTA) prototype (Department of Electrical Engineering and Computer Science and Research Laboratory of Electronics, Massachussetts Insitute of Technology, Cambridge, MA). **(A1)** 3 x 3 mm OCT angiogram of the outer retina with manual removal of the retinal vessel ghost artifact. A sea-fan appearing CNV is seen. **(A2)** Corresponding OCT b-scan showing a retinal pigment epithelial detachment, disruption of Bruch’s membrane, and hyper-reflective material characteristic of CNV. **(B)** The left eye of a 70 year old Caucasian man with treatment-naïve choroidal neovascularization (CNV) due to neovascular age-related macular degeneration (AMD) using the swept source OCTA prototype (Department of Electrical Engineering and Computer Science and Research Laboratory of Electronics, Massachussetts Insitute of Technology, Cambridge, MA). **(B1)** 3 x 3 mm OCT angiogram of the outer retina with manual removal of the retinal vessel ghosting artifact. A sea-fan appearing CNV is seen. **(B2)** Red-free fundus photo exhibiting a lesion of the same shape and location as the CNV seen in B1.

Several publications concerning OCTA of eyes with wet AMD appear in the literature. In July 2014 Jia *et al.* first described the ability of a prototype SS-OCTA system to visualize and quantify CNV that had been seen on FA in five eyes [19]. Then in November 2014, Moult and Choi *et al.* described CNV in 16 of 19 eyes with neovascularization, noting that the majority of these eyes (14/16, 88%) also demonstrated choriocapillaris alteration surrounding the CNV [20]. De Carlo *et al.* described qualitative and quantitative characteristics of CNV in 48 eyes [21]. The group determined sensitivity and specificity of the prototype AngioVue software, using FA as the ground truth, to be 50% (4/8) and 91% (20/22) respectively, hypothesizing that the low sensitivity was due to small sample size and blockage from large amounts of retinal hemorrhage in some patients. Figures 7 and 8 illustrate three examples of CNV, including one type 3 CNV (retinal angiomatous proliferation, RAP), on OCTA confirmed with FA/ICGA, using the Angiovue OCTA software of the RTVue XR Avanti (Optovue, Inc., Fremont, CA). Figure 9 shows two OCTA examples of CNV, one of which was treatment naïve, using the SS-OCT prototype (Department of Electrical Engineering and Computer Science and Research Laboratory of Electronics, Massachussetts Insitute of Technology, Cambridge, MA).

### OCTA of diabetes

There are few published papers as of early 2015 on OCTA of diabetic retinopathy. Choi *et al.* (unpublished data) demonstrated that OCTA of diabetic eyes ranging from no retinopathy to proliferative diabetic retinopathy (PDR) demonstrated choriocapillaris abnormalities and/or retinal microvascular abnormalities such as microaneurysms, vascular remodeling adjacent to the foveal avascular zone (FAZ), enlarged FAZ, and capillary tortuosity and dilation. OCTA and FA were compared in unpublished data by Salz *et al*. The group supported the utility of OCTA in evaluating FAZ and the perifoveal intercapillary area, showing that they were sequentially enlarged in each stages of diabetic retinopathy (normal eyes to PDR). The data showed that OCTA visualized the majority but not all of the microaneurysms visualized by FA likely because OCTA is limited by the principle of slowest detectable flow. However, OCTA was able to appreciate some microaneurysms that were not detected by FA. OCTA also successfully detected other abnormalities that were not evident on FA such as areas of retinal non-perfusion, reduced capillary density, and increased vessel tortuosity. de Carlo *et al.* (unpublished data in review) described a wide-field OCTA montage of an eye with newly proliferative diabetic retinopathy. The wide-field montage OCTA image also successfully allowed visualization of an enlarged FAZ, perifoveal intercapillary area, and multiple microaneurysms. It also provided a larger field of view allowing more peripheral detection of microvascular changes, early NVE, and areas of capillary non-perfusion including areas too small to visualize on FA.

**Figure 10:**
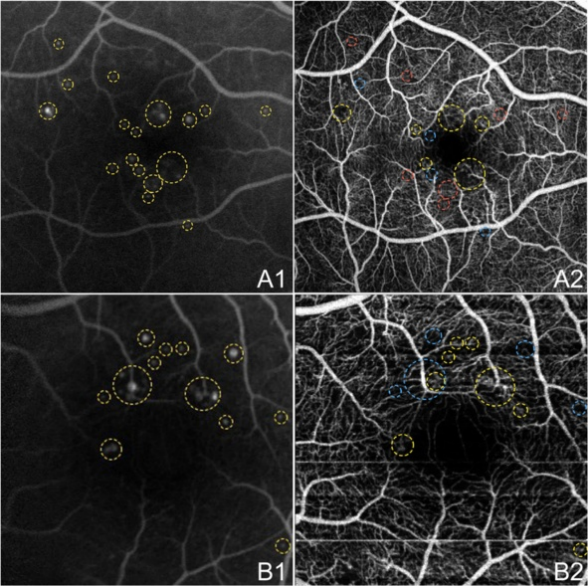
**OCTA and FA of Microaneurysms in NPDR.** The right eye **(A)** and left eye **(B)** of a 45 year old Caucasian man with non-proliferative diabetic retinopathy using the swept source optical coherence tomography angiography (OCTA) prototype (Department of Electrical Engineering and Computer Science and Research Laboratory of Electronics, Massachussetts Insitute of Technology, Cambridge, MA). **(A1)** Fluorescein angiography (FA) cropped to approximately 6 x 6 mm. Aneurysms are circled in yellow. **(A2)** Full-thickness (internal limiting membrane to Bruch’s membrane) 6 x 6 mm OCT angiogram. FAZ appears enlarged. Aneurysms that are seen on FA in A1 that are also seen on OCTA are circled in yellow. Aneurysms on FA that are seen as areas of capillary non-perfusion on OCTA are circled in blue. Areas where aneurysms are seen on FA, but show normal vasculature on OCTA are circled in red. **(B1)** FA cropped to approximately 3 x 3 mm. Aneurysms are circled in yellow. **(B2)** Full-thickness 3 x 3 mm OCT angiogram, which provides improved detail over 6 x 6 mm OCT angiograms, demonstrates higher sensitivity in detecting micro vascular abnormalities. FAZ appears enlarged. Aneurysms that are seen on FA in B1 that are also seen on OCTA are circled in yellow. Aneurysms on FA that are seen as areas of capillary non-perfusion on OCTA are circled in blue.

**Figure 11:**
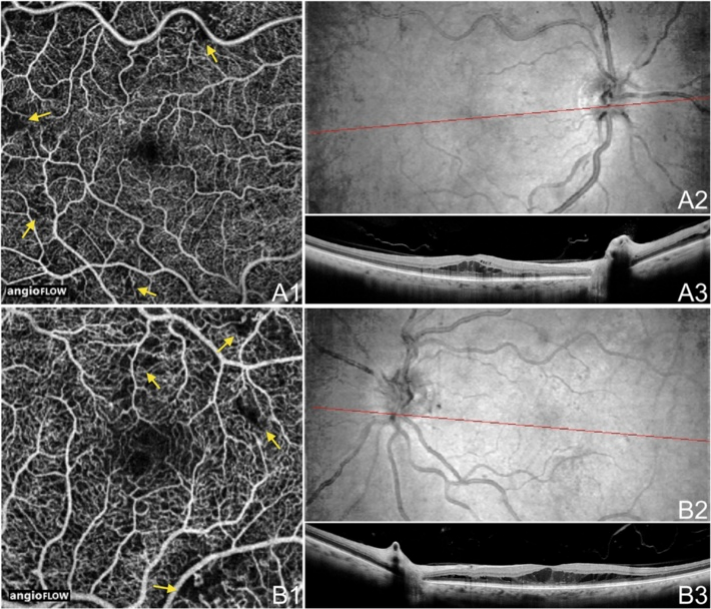
**OCTA of NPDR.** The right eye **(A)** and left eye **(B)** of a 58 year old Caucasian man with non-proliferative diabetic retinopathy and diabetic macular edema (DME) using the Angiovue optical coherence tomography angiography (OCTA) software of the RTVue XR Avanti (Optovue, Inc., Fremont, CA). **(A1)** Full-thickness (internal limiting membrane to Bruch’s membrane) 6 x 6 mm OCT angiogram shows microvascular abnormalities such as areas of capillary non-perfusion (yellow arrows), capillary loops, and microaneurysms. **(A2)** En-face structural OCT with a red line corresponding to the highly-sampled OCT b-scan in A3. **(A3)** 12 mm highly sampled OCT b-scan through the fovea demonstrating DME and hard exudates. **(B1)** Full-thickness 3 x 3 mm OCT angiogram, which provides improved detail over 6 x 6 mm OCT angiograms, shows microvascular abnormalities such as areas of capillary non-perfusion (yellow arrows), capillary loops, and microaneurysms. **(B2)** En-face structural OCT with a red line corresponding to the highly-sampled OCT b-scan in B3. **(B3)** 12 mm highly sampled OCT b-scan through the fovea demonstrating DME and hard exudates.

**Figure 12:**
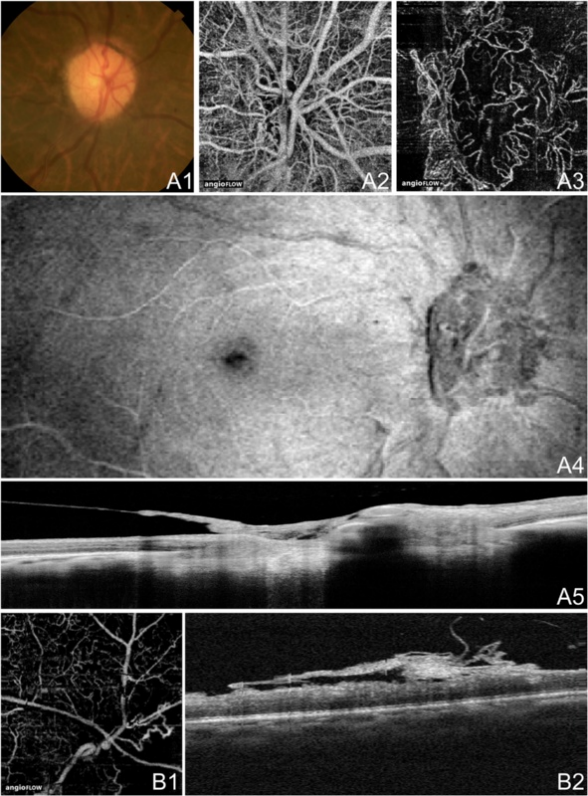
**OCTA of Neovascularization in PDR. (A)** The right eye of a 74 year old African woman with neovascularization of the disc (NVD) due to proliferative diabetic retinopathy (PDR) using the Angiovue optical coherence tomography angiography (OCTA) software of the RTVue XR Avanti (Optovue, Inc., Fremont, CA). **(A1)** Color fundus photo demonstrating fine neovascular vessels over the optic disc. **(A2)** Full-thickness (internal limiting membrane to Bruch’s membrane) 3 x 3 mm OCT angiogram at the optic disc. Wispy NVD is difficult to appreciate. **(A3)** 3 x 3 mm OCT angiogram at the optic disc segmented with the inner boundary in the vitreous above the NVD and the outer boundary slightly below the internal limiting membrane (ILM). The NVD is clearly appreciable. **(A4)** En-face structural OCT showing abnormal tissue at the optic disc. **(A5)** Highly-sampled OCT b-scan of the optic disc where abnormal tissue is observed extending above the ILM into the vitreous cavity. **(B)** The right eye of a 46 year old African woman with neovascularization elsewhere (NVE) due to proliferative diabetic retinopathy (PDR) using the Angiovue optical coherence tomography angiography (OCTA) software of the RTVue XR Avanti (Optovue, Inc., Fremont, CA). **(B1)** 3 x 3 mm OCT angiogram with the inner boundary in the vitreous and the outer boundary at Bruch’s membrane. Abnormal vessels are seen in an area of capillary non-perfusion. Image quality is limited by artifact from movement (horizontal and vertical lines). **(B2)** Corresponding OCT b-scan showing abnormal tissue above the ILM extending into the vitreous cavity.

Figure 10 shows an enlarged FAZ on OCTA and compares OCTA and FA in the identification of microaneurysms in two eyes with non-proliferative diabetic retinopathy (NPDR). Capillary non-perfusion and other retinal microvascular abnormalities are demonstrated in Figure 11. OCTA examples of NVD and NVE in PDR eyes are shown in Figure 12.

### OCTA of artery and vein occlusion

Retinal vascular occlusions have yet to be described in the literature using OCTA as an imaging modality. However, preliminary work at the New England Eye Center of Boston, MA shows that OCTA may be useful for evaluating these diseases. Unpublished data in review by de Carlo *et al.* described a case of branch retinal vein occlusion (BRVO) using a wide-field montage technique. The OCTA showed a large wedge-shaped area of capillary non-perfusion in the inferotemporal macula with clear delineation of the boundary of ischemia, and vascular abnormalities such as microaneurysms, telangiectasis, and anastamoses.

**Figure 13:**
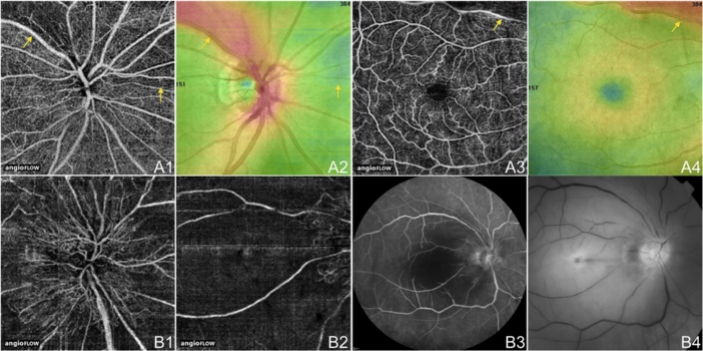
**OCTA of BRAO and CRAO. (A)** The right eye of a 70 year old Caucasian man with an acute branch retinal artery occlusion using the Angiovue optical coherence tomography angiography (OCTA) software of the RTVue XR Avanti (Optovue, Inc., Fremont, CA). **(A1)** Full-thickness (internal limiting membrane to Bruch’s membrane) 4.5 x 4.5 mm OCT angiogram of the optic disc showing decreased capillary perfusion superotemporal and nasal to the disc (yellow arrows). **(A2)** 4.5 x 4.5 mm en-face OCT thickness map showing retinal thickening in red and thinning in blue (yellow arrows) that correspond to the decreased capillary perfusion in A1. **(A3)** Full-thickness 6 x 6 mm OCT angiogram illustrating decreased capillary perfusion superotemporal and nasal to the disc (yellow arrow) as in A1. **(A4)** 6 x 6 mm en-face OCT thickness map showing retinal thickening in red (yellow arrow) that correspond to the decreased capillary perfusion in A3. **(B)** The right eye of an 81 year old Caucasian man with a subacute central retinal artery occlusion using the Angiovue optical coherence tomography angiography (OCTA) software of the RTVue XR Avanti (Optovue, Inc., Fremont, CA). **(B1)** Full-thickness 4.5 x 4.5 mm OCT angiogram of the optic disc showing diffusely decreased peripapillary capillary perfusion. **(B2)** Full-thickness 6 x 6 mm OCT angiogram illustrating decreased capillary perfusion in the macula. Only the large retinal and peripapillary vessels demonstrate blood flow. **(B3)** Fluorescein angiography is hypo-fluorescent in the macula and peripapillary region due to the decreased ability for the fluorescein dye to reach these areas because of low blood flow. The vessels appear attenuated. **(B4)** Red-free fundus photo demonstrates attenuation of the vessels especially in the macular and peripapillary regions.

Figure 13 shows OCT angiograms of an acute branch retinal artery occlusion (BRAO) and a subacute central retinal artery occlusion (CRAO). The BRAO demonstrates wedge-shaped areas of capillary non-perfusion that correlate to areas of abnormalities on the retinal thickness map. This illustrates the potential use of OCTA in pinpointing areas of ischemia and edema. The CRAO shows diffuse capillary non-perfusion in areas supplied by the central retinal artery as seen on the same-day FA. Flow is still seen in the major retinal vessels. Around the optic disc, there is an absence of blood flow in the superficial disc vasculature supplied by the central retinal artery but the lamina cribosa blood flow remains intact. As OCTA provides a snapshot in time, it does not demonstrate delayed arteriovenous transit time as FA does.

**Figure 14:**
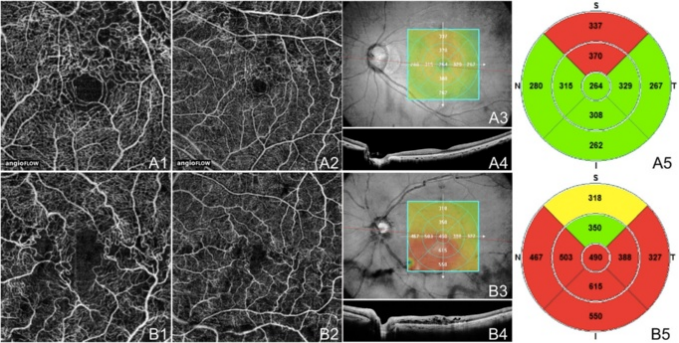
**OCTA of BRVO and CRVO. (A)** The left eye of a 61 year old Asian woman with a chronic branch retinal vein occlusion using the Angiovue optical coherence tomography angiography (OCTA) software of the RTVue XR Avanti (Optovue, Inc., Fremont, CA). **(A1)** Full-thickness (internal limiting membrane to Bruch’s membrane) 3 x 3 mm OCT angiogram showing capillary non-perfusion superotemporal extending into the foveal avascular zone (FAZ) and telangiectatic vessels at the border of the ischemic areas. **(A2)** Full-thickness 6 x 6 mm OCT angiogram demonstrating that the capillary non-perfusion is along the superior arcade. The edges of the ischemia are bordered by telangiectatic vessels, capillary loops, and possible microaneurysms. **(A3)** En-face structural OCT with a retinal thickness map and a red line corresponding to the highly-sampled OCT b-scan in A4. **(A4)** 12 mm highly sampled OCT b-scan through the fovea which appears relatively unaffected. **(A5)** Retinal thickness map demonstrating superior thickening due to edema. **(B)** The left eye of a 72 year old Caucasian man with a chronic central retinal vein occlusion using the Angiovue OCTA software of the RTVue XR Avanti (Optovue, Inc., Fremont, CA). **(B1)** Full-thickness 3 x 3 mm OCT angiogram showing diffuse capillary non-perfusion continuous with the FAZ and telangiectatic vessels. **(B2)** Full-thickness 6 x 6 mm OCT angiogram demonstrating telangiectatic vessels and diffuse capillary non-perfusion especially along the inferior arcade. **(B3)** En-face structural OCT with a retinal thickness map and a red line corresponding to the highly-sampled OCT b-scan in B4. **(B4)** 12 mm highly sampled OCT b-scan through the fovea which shows macular edema and disruption of the photoreceptor layer. **(B5)** Retinal thickness map demonstrating thickening that is greatest inferiorly.

A case of BRVO and a case of central retinal vein occlusion (CRVO) are illustrated in Figure 14. OCTA of the BRVO shows capillary non-perfusion superotemporally along the superior arcade extending into the FAZ, and telangiectatic vessels, capillary loops, and possible microaneurysms at the border of the ischemic areas. The OCTA of the chronic CRVO demonstrates diffuse capillary non-perfusion continuous with the FAZ and telangiectatic vessels.

### OCTA of glaucoma

OCTA is a useful tool for evaluating optic disc perfusion in glaucomatous eyes. The normally dense peripapillary microvascular network is attenuated in both the superficial disc vasculature and the deeper lamina cribosa. Averaging the decorrelation signal in OCT angiograms approximates the area of microvasculature and allows the user to calculate the flow index, which is decreased in eyes with glaucoma. The flow index has been shown to have both a very high sensitivity and specificity in differentiating glaucomatous eyes from normal eyes [22,23].

## Conclusions

OCTA is a new technology that has great potential for use in the clinical setting. Compared with FA and ICGA, the current retinal angiographic gold standards, OCTA advantages are that it is non-invasive, acquires volumetric scans that can be segmented to specific depths, uses motion contrast instead of intravenous dye, can be obtained within seconds, provides accurate size and localization information, visualizes both the retinal and choroidal vasculature, and shows structural and blood flow information in tandem. Disadvantages of OCTA are its limited field of view, inability to view leakage, increased potential for artifacts (blinks, movement, vessel ghosting), and inability to detect blood flow below the slowest detectable flow.

OCTA has been shown to be a useful imaging modality for the evaluation of common ophthalmologic diseases such AMD, diabetic retinopathy, artery and vein occlusions, and glaucoma. In some cases OCTA has even been shown to detect pathology not seen on FA. In the future, faster scanning speeds would be crucial to obtain larger fields of view with higher resolution. More studies are needed to determine OCTA’s utility in the clinical setting and to determine if this technology may offer a non-invasive option of visualizing the retinal vasculature in detail.

## Abbreviations

AMD: Age-related macular degeneration
BRAO: Branch retinal artery occlusion
BRVO: Branch retinal vein occlusions
CRAO: Central retinal artery occlusion
CRVO: Central retinal vein occlusions
CSCR: Central serous chorioretinopathy
CNV: Choroidal neovascularization
FA: Fluorescein angiography
FAZ: Foveal avascular zone
GCL: Ganglion cell layer
GA: Geographic atrophy
ICGA: Indocyanine green angiography
INL: Inner nuclear layer
IPL: Inner plexiform layer
ILM: Internal limiting membrane
NVE: Neovascularization elsewhere
NVD: Neovascularization of the disc
NPDR: Non-proliferative diabetic retinopathy
OCT: Optical coherence tomography
OCTA: Optical coherence tomography angiography
OPL: Outer plexiform layer
RPED: Retinal pigment epithelial detachment
PCV: Polypoidal choroidal vasculopathy
PDR: Proliferative diabetic retinopathy
RAP: Retinal angiomatous proliferation
RNFL: Retinal nerve fiber layer
RPE: Retinal pigment epithelium
SD-OCT: Spectral domain optical coherence tomography
SSADA: Split-spectrum amplitude decorrelation angiography
SS-OCT: Swept source optical coherence tomography
VCSEL: Vertical cavity surface emitting laser
